# 

**Published:** 1887-04

**Authors:** 


### CONTENTS.

### ORIGINAL COMMUNICATIONS—

An Address to the Graduating Class of the Atlanta Medical College. By Henry Clay Morrison, D. D., Atlanta, Ga.......... 65

On Certain Medico-Legal Subjects. By J. J. Abrams, Esq., Savannah, Ga.......... 74

Remittent Fever—its Prompt Arrest and Effective Treatment. By John L. Jones, M. D., New Orleans, La.......... 83

### CLINIC REPORTS—

Calcification of Blood-Vessels Following Injury. By W. F. Westmoreland, Jr., M. D., Atlanta, Ga.......... 88

### SOCIETY REPORTS—

Clinical Society of Maryland.......... 90

### CORRESPONDENCE—

Our New York Letter.......... 96

Taliaferro and Bozeman Controversy. By V. H. Taliaferro, M. D. 100

Ninth International Medical Congress.......... 102

### BOOK REVIEWS—

A Text-book of Surgery. By J. A. Wyeth.......... 103

Medical and Surgical Memoirs. By Joseph Jones.......... 104

A Reference Hand-book of the Medical Sciences. William Wood & Co.......... 107

On Diseases of the Lungs and Pleuræ, Including Consumption. Powell.......... 110

Nervous Diseases and their Diagnosis. H. C. Wood.......... 110

A Treatise on Compound Ophthalmic Lenses. Prentice.......... 112

### EDITORIAL—

Confidential Relations Between Physician and Patient.......... 113

The Medical Association of Georgia.......... 114

Thou Shalt Not Steal.......... 116

Appropriation for the Investigation of Inoculation against Yellow Fever.......... 117

Death of Dr. John G. Westmoreland.......... 119

Items.......... 222

Our Advertisers.......... 128

